# Coordination mechanism of construction industry supply chain based on data elements

**DOI:** 10.1371/journal.pone.0323748

**Published:** 2025-05-20

**Authors:** Hong Zhang

**Affiliations:** School of Economics and Management, Beijing Jiaotong University, Beijing, P.R.China; The University of Alabama, UNITED STATES OF AMERICA

## Abstract

This paper presents a comprehensive approach to enhancing the coordination of the construction industry supply chain by incorporating data elements into the decision-making process. A two-level supply chain model is developed, consisting of a core enterprise and suppliers, initially analyzed in a non-contractual environment. The findings reveal inefficiencies in supply chain coordination under these conditions, prompting the design of a cost-sharing and profit-sharing contract model. By applying these contracts, supply chain coordination is significantly improved, leading to enhanced collaboration, optimized resource allocation, and increased profitability for all stakeholders. The innovative contribution of this research lies in its integration of data elements with contractual coordination mechanisms, which is verified through practical case studies and numerical simulations, offering a novel perspective and methodology for managing construction industry supply chains.

## 1. Introduction

The construction supply chain is not a chain of construction enterprises with business-to-business relationships, but a network composed of multiple organizations and relationships, including the flow of information, materials, services, or products, as well as financial flows among customers, core enterprises, suppliers, banks, and regulatory authorities. In construction projects, the complexity of the supply chain is evident, with various factors such as project scale, time constraints, liquidity of funds, and market volatility posing severe challenges to supply chain management. Through collaborative decision-making, all parties can share resources, information, and technology, reduce unnecessary communication costs and misunderstandings, and better utilize resources to advance the project.

The most direct conflict is the supply price between suppliers and core enterprises. Core enterprises aim to procure materials and services at a lower cost to increase profit margins, while suppliers wish to sell their products and services at higher prices to maximize their own returns. Core enterprises tend to extend payment terms to maintain cash flow flexibility, whereas suppliers usually desire prompt payment to alleviate their financial pressure.

Core enterprises may seek more lenient loan conditions (less regulation, lower collateral requirements, and longer repayment periods), while banks, for risk control purposes, may set stricter loan conditions. Core enterprises hope to obtain low-cost financing to support their projects, while banks focus on generating profits and compensating for risk through higher interest rates and fees.

To achieve a dynamic balance of collaborative interests among supply chain enterprises, a win-win premise is necessary. If core enterprises overly encroach on suppliers’ interests, it may lead to opportunistic behaviors such as cutting corners or substituting inferior products, ultimately harming the core enterprise’s interests. If suppliers provide false progress information to obtain funds early, it may cause core enterprises to arrange subsequent work based on unrealistic progress, leading to overall project delays, further increasing project costs, and potentially facing default risks. Meanwhile, if banks release funds based on false progress, it may lead to inefficient use of funds or even the misuse or misappropriation of funds, thus increasing the bank’s financial risk.

In an environment without formal contracts, the lack of clear contractual terms can lead to ambiguity in risk allocation and responsibility delineation within the supply chain. This uncertainty may result in issues with liquidity, affecting the efficiency and effectiveness of the entire supply chain. In the coordination of supply chain financial operation decisions without contracts, the Stackelberg game model is used, which sets the core enterprise as the leader and the suppliers as followers (responding to the leader’s decisions). The main process includes: the core enterprise formulates its decisions based on its understanding of project requirements and assessment of the supply chain environment, such as setting project budgets and determining payment terms. Suppliers or banks then develop their strategies based on the core enterprise’s decisions. For example, suppliers can decide on the quantity of goods supplied and their pricing, while banks determine the loan amount and interest rate.

This paper selects the Stackelberg model over the Nash equilibrium or cooperative game models for the following reasons:The Nash equilibrium assumes simultaneous decision making by all participants and symmetric information, which is uncommon in real world supply chains. In contrast, the Stackelberg model, by introducing leader follower roles, can more accurately simulate actual supply chain decision making processes.

Cooperative game models assume full cooperation and information sharing among participants. However, in non-contractual settings, this assumption is overly idealistic. The Stackelberg model, not relying on assumptions of complete cooperation and information sharing, is better suited for non cooperative scenarios.

Given its suitability for non-contractual environments, this paper employs the Stackelberg game model to investigate construction industry supply chain financing coordination.

The Stackelberg model has been effectively applied in various scenarios, such as resource allocation and intelligent scheduling. For instance, in the smart scheduling of electric - vehicle charging stations, operators (as leaders) set pricing strategies, and users (as followers) choose optimal charging times. Similarly, in the construction - industry supply chain, core enterprises can act as leaders to establish contract terms, while suppliers, acting as followers, adjust their production strategies accordingly.

Contracts have traditionally been used as a means to coordinate buyers and suppliers in the supply chain to achieve higher system efficiency. There are two types of contracting: traditional contracting and relational contracting. In traditional contracts, the identity and individual attributes of the parties are irrelevant, and these contracts do not emphasize coordination, lacking in considerations of the needs for ongoing relationships, collaborative and cooperative management, or risks and uncertainties. In contrast, in relational contracts, the identity and individual attributes of the parties are crucial, and written documents are seen as records of consensus, which can promote coordination among the parties in the supply chain. Moreover, the completeness of the contract greatly impacts the coordination of the construction industry supply chain. However, in most cases, construction contracts are inherently incomplete.

Supply chain contracts are divided into the following four main types: wholesale price contract, buy-back contract, revenue sharing contract, and quantity discount contract. Among these, wholesale price contracts and buy-back contracts are the earliest studied and most common types, while revenue sharing contracts and quantity discount contracts focus on the two core aspects of the supply chain: the profits of the participating parties and the product quantity. Of course, in addition to these four contract models, there are many others, such as quantity flexibility contracts, quantity commitment contracts, option contracts, pay to delay contracts, advance purchase contracts, and rebate and penalty contracts[1]. However, these contract models can all be evolved from the aforementioned four types, or they can be combinations of two or more of these contracts. To achieve effective supply chain coordination, both parties to the contract need to recognize the importance of completeness and relational dimensions. While achieving an ideal state of contract completeness is difficult, transparency and mutual understanding during contract execution can be improved through appropriate mechanisms and strategies, such as regular communication meetings, contract reviews and adjustments, and clear principles of risk sharing.

Among them, revenue sharing contracts allow parties in the supply chain to share the ultimate revenue, encouraging cooperation and resource sharing among partners. In a revenue sharing contract, participants in the supply chain (such as manufacturers and retailers) agree to share the total project revenue according to a certain proportion. The core enterprise and the supplier negotiate the revenue-sharing ratio based on expected sales volume and production costs, and the supplier makes production decisions based on engineering order information and production costs, while the core enterprise makes planning decisions based on project requirements to maximize the total revenue of the entire supply chain, while ensuring that the earnings of each party meet their expected levels.

Cost-sharing contracts involve participants in the supply chain jointly bearing a portion of the project costs to alleviate the financial burden of a single participant and to promote effective resource allocation and risk sharing. The core enterprise and the supplier negotiate the specific proportion and conditions for cost sharing, and based on the shared ratio, they jointly decide the project’s investment scale and capital allocation. When implementing construction projects, revenue distribution and cost compensation are carried out based on actual costs and benefits to maximize the total profit of the supply chain while managing cost risks and investment returns.

This paper discusses the modelling of construction industry supply chain financing coordination under two conditions, without and with contract, respectively, based on the premise of data elements. How to coordinate the construction industry suppliers’ investment level, pricing and supply chain profits in the case of adopting no contract; and analyze the sensitivity of its effect of coordinating optimal decisions and equilibrium outcomes to the overall supply chain after adopting no revenue-sharing contract, and draw conclusions through numerical analysis. In the contractual case, the coordination model of supply chain financing decisions under the revenue sharing contract and cost sharing contract is established to analyze the sensitivity of the effect of its coordination of optimal decisions and equilibrium outcomes on the overall supply chain after the adoption of the contract; conclusions are drawn through numerical analysis.

Chapter 1 of this paper introduces the background of supply chain financing coordination in the construction industry, explains the supply chain financing coordination in the construction industry with and without contractual environment, and paves the way for the later problem development and modelling. Chapter 2 provides an overview of relevant studies on supply chain financing coordination in the construction industry at home and abroad in recent years. Chapter 3 presents the main research questions and models the two scenarios of supply chain coordination in the construction industry by combining the data elements, and establishes the cost-sharing contract, benefit-sharing contract, and cost-sharing-benefit-sharing contract models, respectively. Chapter 4 analyses the proposed models numerically, using real data to test the validity and parameter sensitivity of the models. Finally, Chapter 5 concludes that it is necessary to establish relevant covenants to ensure the distribution of benefits, and also proposes an outlook for further research.

## 2. Literature review

The construction industry, as an important part of the national economy, has always drawn attention to the issue of supply chain financing coordination. Scholars both domestically and internationally have made progress in researching the coordination of construction industry supply chain financing.

Firstly, scholars have focused on the relationships between core enterprises, suppliers, and financial institutions in the construction industry supply chain, exploring challenges and solutions in terms of capital flows and information sharing. By analyzing various conflicts and cooperation within the construction industry supply chain, researchers have put forward a series of recommendations to promote the development of construction industry supply chain financing coordination.

Using methods such as establishing mathematical models, applying game theory, and coordination theory, they have explored supply chain coordination mechanisms under different contract forms. The impact of cooperative approaches such as cost-sharing and revenue-sharing on supply chain efficiency has been analyzed, and some feasible suggestions and solutions have been proposed.

Ding et al.[2] explored the financing decisions of suppliers and the coordination of the supply chain under conditions of uncertain output. To study the optimal financing strategy in the distribution channel, Chen et al.[3] compared and analyzed the expected profits of retailers and suppliers under bank credit financing and trade credit financing, respectively. Jing et al.[4] demonstrated that under the trade credit financing method, retailers and manufacturers share the demand risk, thereby incentivizing retailers to increase their inventory levels. Cai et al.[5] analyzed the optimal decisions under single credit and trade-bank credit from the retailer’s perspective, and further studied the complementarity and substantive relationship between bank credit and trade credit. Peng et al.[6] introduced the concept of expected interest rate and compared the optimal decisions under the advance payment mechanism with those under bank financing.

Furthermore, there is also a focus on the application of digital technologies in the coordination of construction industry supply chain financing. Researchers have studied how to leverage artificial intelligence, big data, and other technological methods to optimize the management and decision-making of the construction industry supply chain, thereby enhancing the efficiency and coordination of the supply chain.

Rosenman et al.[7] developed a virtual collaborative environment by connecting BIM elements across different disciplines, enabling the integration of BIM information across disciplines. Chen et al.[8] developed a BIM data server based on IFC to support architects and structural engineers in collaborative design over the web. Currently, some commercial collaborative work platforms, such as Buzzsaw, A-site, 4Projects, and Onuma, are beginning to support BIM, and intelligent control platforms for construction industry supply chain coordination are also being applied.

Overall, researchers both domestically and internationally have achieved comprehensive coverage from theoretical research to practical cases in the field of construction industry supply chain financing coordination, providing a solid theoretical foundation and practical guidance for the development of domestic construction industry supply chain management. It is hoped that scholars from home and abroad can strengthen cooperation and exchange in the future, jointly promote the research and practice of construction industry supply chain financing coordination, and make greater contributions to the sustainable development of the construction industry and the improvement of global supply chain management levels.

## 3. Problem definition

### 3.1. Problem description

**Fig 1:**
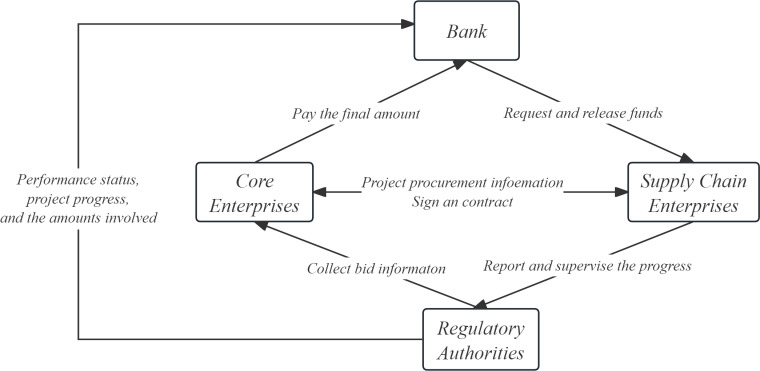
Block diagram of construction supply chain operation process based on data elements.

In the supply chain of the construction industry, upstream suppliers conduct building materials production, raw material procurement, product design, etc. according to the winning contract, and provide the building materials to the core enterprises. The core enterprises carry out construction, and the core enterprises finally sell the finished products to customers. This paper considers a construction supply chain composed of a core enterprise and a supplier, who are directly selected by the core enterprise and managed according to the business contract. In the absence of contract, the core enterprises and suppliers, as independent individuals, make independent decisions and aim to maximize their own profits. In the case of revenue sharing and cost sharing, the core enterprises and suppliers aim to maximize the overall profit of the construction supply chain. Fig 1 is a block diagram of the construction supply chain operation process based on data elements, from which the role of data elements in each link of the supply chain can be seen.

In the Block diagram of this construction supply chain, six main processes can be seen, including:

1. The core enterprise releases project procurement information, and supply chain enterprises participate in the bidding: After determining project requirements, the core enterprise releases detailed procurement information. This may include expected construction plans, necessary equipment and material lists, expected project timelines, etc. At the same time, the core enterprise will set a bid date, allowing various supply chain enterprises to understand the requirements and prepare for the bid. Each supply chain enterprise will plan detailed proposals and price quotations based on the information released by the core enterprise. The information they need to submit may include, but is not limited to: basic company information, experience in executing similar projects in the past, expected project implementation plans, detailed cost and price quotations, and other related operational information. During this process, the regulatory authorities will verify the authenticity of the information, collect bid information, and verify the information of the supply chain enterprises. They may conduct credit checks, historical performance reviews, and price reviews on the bidders. Only when the stamping authority confirms that the information is authentic and compliant will the enterprise enter the next round of the bidding process.
2. Signing contracts: After bid evaluation, the core enterprise will select the supply chain enterprise that meets the project requirements and sign a contract with it. The contract will detail the specific requirements of the project, the execution timeline, payment methods, related responsibilities and obligations.
3. Suppliers perform contracts according to the contract: Suppliers begin to perform the contract according to the contract requirements, implement the project plan, and submit construction progress and product lists to the regulatory authorities at specific time points.
4. Suppliers feedback progress: Suppliers submit progress and work quantity related certificates along with financing applications to banks and regulatory authorities. After receiving this information, the regulatory authorities will review it, confirm that there are no errors, and then feedback the information to the core enterprise.
5. Regulatory authorities submit information to the bank: After the regulatory review, the relevant performance and audit reports are submitted to the bank. These reports will include all verification information, including the supplier’s performance status, project progress, and the amounts involved, etc.
6. Banks and core enterprises disburse funds: After confirming no errors, the enterprise waits for the bank’s verification. After verification, the bank will disburse the corresponding funds directly to the supplier according to the contract and the supplier’s performance. After receiving the funds, the supplier will deliver goods according to the contract and project progress. Once the project is fully completed, the core enterprise will pay the final amount to the bank. Thus, the entire cooperation process is completed.

This entire process is carried out under strict supervision and verification to ensure the compliance of all activities, reduce the risk of supply chain finance, and also ensure the smooth progress of the engineering project. Overall, this supply chain finance model fully utilizes the advantages of each participant, optimizes and efficiently operates each link of the supply chain, and provides an enterprise with a cooperation model that meets market demands and modern management requirements.

### 3.2. The impact of data timeliness on system sensitivity

This study assumes symmetric information between core enterprises and suppliers in analyzing construction supply chain coordination mechanisms. However, information asymmetry is common in real - world supply chain management. Core enterprises often have more information on market demand, project progress, and financial status, while suppliers have limited access to such information. This asymmetry can lead to suboptimal supplier decisions, negatively affecting the overall efficiency and coordination of the supply chain.

Data timeliness is crucial for supply chain management in the construction industry. It enables the system to better respond to market demand fluctuations, material cost changes, and external disturbances. Accurate and timely data can significantly reduce supply chain sensitivity, enhancing its stability and adaptability, and improving overall efficiency and competitiveness.

1. Impact on Sensitivity to Market Demand Changes

Market demand is highly uncertain and volatile in the construction industry. Timely market data, such as bidding information, real estate trends, and infrastructure plans, allows supply chain participants to anticipate market movements and adjust production and procurement strategies. For example, core enterprises can quickly increase raw material procurement and arrange construction teams when demand surges, or reduce inventory buildup when demand declines. Lagging data can lead to slow responses, causing over - or under - supply, increased costs, and customer dissatisfaction.

2. Impact on Sensitivity to Material Costs

Fluctuations in building material costs directly affect profit margins. Timely material price data, supplier cost structures, and logistics cost changes help with cost control and optimization. For instance, core enterprises can pre - negotiate with suppliers to adjust procurement strategies when major material prices rise, such as steel or cement. They can sign short - term contracts to lock in prices or find alternatives. Suppliers can also adjust production and pricing strategies based on cost changes. Untimely data updates can result in procurement decision errors, leading to unnecessary high costs and reduced profits.

3. Impact on Sensitivity to External Disturbances

Construction supply chains face various external disturbances, including natural disasters, regulatory changes, and public health events. Timely monitoring and early warning of external environmental data can help the supply chain system prepare in advance and mitigate negative impacts. For example, core enterprises and suppliers can adjust construction schedules and stockpile materials before typhoon season based on weather forecasts. They can also quickly adapt to new policies such as environmental requirements or quality standards to ensure compliance and avoid operational risks.

In conclusion, this paper highlights the importance of data timeliness in reducing system sensitivity and promoting information sharing among supply chain enterprises. Establishing information - sharing platforms and achieving real - time data sharing through digital means can enhance supply chain stability and competitiveness. For instance, the “Construction Cloud” platform used in the Chengdu Tianfu International Airport Terminal Area project enabled electronic storage and real - time updates of procurement data, allowing suppliers to access the latest project requirements and progress information. Regular communication meetings and information bulletins can also ensure suppliers are promptly informed of core enterprise decisions and market dynamics. Additionally, including information - sharing terms and responsibilities in contracts, and involving third - party regulatory or auditing institutions to ensure information transparency and authenticity, can further strengthen supplier trust in core enterprises.

By simulating system responses under different data update frequencies, a quantitative relationship between data timeliness and supply chain stability can be established. Higher data update frequencies result in lower system sensitivity to changes, smaller fluctuations in indicators, and stronger stability. In summary, timely data updates can reduce the uncertainty of market environmental fluctuations for the entire system. However, to simplify the analysis, this paper excludes the impact of market environmental changes such as market demand fluctuations, material cost changes, and external disturbances, and only explores supply chain financing decision coordination by adjusting the revenue - sharing ratio.

### 3.3. Symbol description and basic assumptions

#### 3.3.1. Symbol description.

In the following section, we provide an explanation of the key symbols used in the mathematical models throughout this paper. These symbols represent the various parameters and variables necessary for defining the construction supply chain’s operational and financial dynamics.

Table 1 shows the model symbols and explanations of the symbols to set the stage for the modeling below.

**Table 1:** Model symbols and explanations.

Model symbol	Explanation
c	Unit product cost of the supplier
w	Sales price per unit product of the supplier
p	Sales price per unit product of the core enterprise
$c_{u}$	Contractor’s shortage loss per unit of the core enterprise
v	Residual value of materials remaining per unit after completion of project
Q	Order quantity of core enterprise
x	Requirements for engineering materials
F(x)	Cumulative distribution function of material demand
f(x)	Probability density function of material demand
u	Mean material demand
s(q)	Expected use of materials in the core enterprises
r	Income distribution ratio

In general, in reality, the sales price of the unit product of the core enterprise is greater than the unit sales price of the unit product, the unit product sales price of the supplier is greater than the unit product cost, and the unit product cost is greater than the residual value of the unit remaining material after the completion of the project, it is concluded that p > w > c > v.

#### 3.3.2. Basic assumptions.

To facilitate the study, this chapter makes the following assumptions:

1) The core enterprise is in the leading position, and the suppliers are the followers;
2) The core enterprise and supplier are risk-neutral and completely rational, and the information of both parties is symmetrical, that is, both parties understand all the information of both parties, so as to make the best decision;
3) Without considering the capacity constraints, that is, the production capacity of the supplier is unlimited, excluding the inventory cost;
4) The contractor’s demand for materials depends on the project demand, which has a differentiable distribution function and a differentiable and invertible probability density function.

#### 3.3.3. Model building and solution.

The expected material use of the core enterprises is determined jointly by the product purchase amount and the level of engineering material demand,

When the actual demand is less than/equal to the order quantity, x≤Q


s(q)=x (1)

When the actual demand is more than the order quantity,    x>Q


s(q)=Q (2)

Therefore, the material use amounts of the core enterprise is:


$$s\left(q\right)=E\left(\min\left(x,Q\right)\right)=\int_{0}^{+\infty }f\left(x\right)\min\left(x,Q\right)dx=\int_{0}^{q}\int_{y}^{+\infty }f\left(x\right)dydx$$ (3)

$$s\left(q\right)=Q-\int_{0}^{Q}F\left(x\right)dx$$ (4)

Expected material shortage volume of core enterprise:


$$L\left(q\right)=\int_{q}^{+\infty }\left(x-Q\right)f\left(x\right)dx=\int_{0}^{+\infty }\left(x-Q\right)f\left(x\right)dx-\int_{0}^{Q}\left(x-Q\right)f\left(x\right)dx$$ (5)

$$L\left(q\right)=E\left(x\right)-Q+\int_{0}^{Q}\left(Q-x\right)f\left(x\right)dx=E\left(x\right)-Q+\int_{0}^{Q}f\left(x\right)\left(\int_{x}^{Q}1dy\right)dx$$ (6)

$$L\left(q\right)=E\left(x\right)-Q+\int_{0}^{Q}\int_{0}^{y}f\left(x\right)dxdy=E\left(x\right)-Q+\int_{0}^{Q}F\left(x\right)-F\left(0\right)dx$$ (7)

$$L\left(q\right)=E\left(x\right)-\int_{0}^{Q}1dx+\int_{0}^{Q}F\left(x\right)dx=E\left(x\right)-s\left(Q\right)$$ (8)

Expected material surplus of the core enterprise:


$$\pi_{t}\left(q\right)=ps\left(q\right)+vI\left(q\right)-wQ-c_{u}L\left(q\right)=ps\left(q\right)+vq-vs\left(q\right)-wQ-c_{u}\left(E\left(x\right)-s\left(q\right)\right)$$ (9)

$$\pi_{t}\left(q\right)=\left(p-v+c_{u}\right)s\left(q\right)+\left(v-w\right)Q-c_{u}E\left(x\right)$$ (10)

### 3.4. Study on the coordination of supply chain finance operation decisions without contract

**Fig 2:**
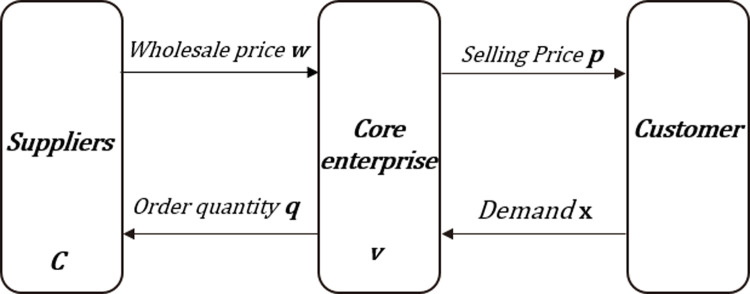
No-contract model of the supply chain.

In the absence of contract, the core contractor enterprise and the supplier enterprise are independent rational economic entities, and they have no subordinate relationship. Both of them pursue the maximization of their own interests. The core enterprises decide the order quantity of goods according to the project demand and the wholesale price, and bear the risk of oversupply. The surplus goods are treated at a discount, and the suppliers produce according to the order quantity of the contractor, without taking the risk of inventory and market demand. **Fig 2 shows the model without a contract.**

The optimal order quantity of the core enterprise is determined by the expected profit function, and the partial derivative of the core enterprise profit function can be derived according to the Leibzny formula:


$$\frac{d\pi_{t}\left(q\right)}{dQ}=\left(p-v+c_{u}\right)*\left(1-F\left(q\right)\right)+v-w=p+c_{u}-w-\left(p-v+c_{u}\right)F\left(q\right)$$ (11)

$$\frac{d^{2}\pi_{t}\left(q\right)}{dQ^{2}}=-\left(p-v+c_{u}\right)f\left(q\right)$$ (12)

f(q)>0,p>v Obviously, so the second order derivative is less than 0, and the expected profit of the core enterprise is a convex function of q, so there is a unique optimal order quantity that maximizes the expected profit.


$$\frac{d\pi_{t}\left(q\right)}{dq}=p+c_{u}-w-\left(p-v+c_{u}\right)F\left(q\right)=0$$ (13)

$${Q_{1}}^{*}=F^{-1}\left(\frac{p+c_{u}-w}{p-v+c_{u}}\right)$$ (14)

Suppliers also determine their own profits according to the optimal order quantity, and can substitute the optimal order quantity into the expected profit function of the core enterprises and suppliers, and the overall profit of the supply chain can be obtained.

From the perspective of supply chain management, the overall expected profit of the supply chain is:


$$\pi \left(q\right)=\pi_{t}\left(q\right)+\pi_{s}\left(q\right)=\left(p-v+c_{u}\right)s\left(q\right)+\left(v-c\right)Q-c_{u}E\left(x\right)$$ (15)

$$\frac{d\pi \left(q\right)}{dQ}=\left(p-v+c_{u}\right)*\left(1-F\left(Q\right)\right)+v-c=p+c_{u}-c-\left(p-v+c_{u}\right)F\left(q\right)$$ (16)

$$\frac{d^{2}\pi_{t}\left(q\right)}{dq^{2}}=-\left(p-v+c_{u}\right)f\left(q\right)<0$$ (17)

$$\frac{d\pi_{t}\left(q\right)}{dq}=p+c_{u}-c-\left(p-v+c_{u}\right)F\left(q\right)=0$$ (18)

$${Q_{2}}^{*}=F^{-1}\left(\frac{p+c_{u}-c}{p-v+c_{u}}\right)$$ (19)

$$\frac{p+c_{u}-c}{p-v+c_{u}}>\frac{p+c_{u}-w}{p-v+c_{u}}$$ (20)

Therefore, at this point the overall benefit of the supply chain is not maximized and the supply chain does not reach the coordination. If you want to achieve coordination, you can only make    c=w,but at this time the core enterprise occupies all the profits, which is impossible in the actual production situation.

### 3.5. Study on supply chain financing decision coordination under cost-sharing contract

**Fig 3:**
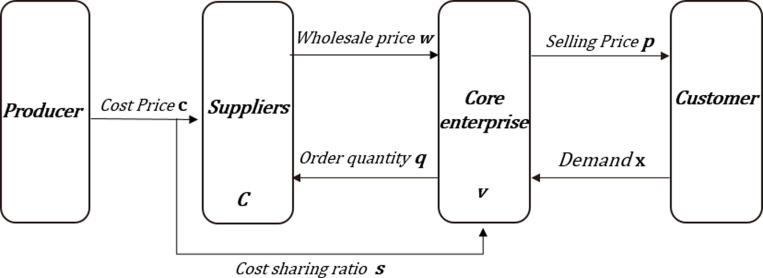
Cost-sharing model of the supply chain.

As shown in Fig 3, the cost-sharing contract model allows costs and risks to be shared between the core business and the supplier. By sharing costs, both parties will build a stronger relationship and have a greater incentive to seek efficiencies and optimizations, to work together to reduce costs across the supply chain and to find better solutions to meet customer needs, thus helping to ensure that the core business and the supplier are on an even keel, while optimizing costs and efficiencies across the supply chain to the fullest extent possible[9]. The core enterprise is still the leader, deciding to bear s proportional of the cost to expect lower wholesale price of building materials and encourage suppliers to invest in the project; the suppliers, as followers, tend to sell materials to the core enterprise at a lower price, and establish long-term cooperative relationship with the core enterprise to attract more orders.

For the core enterprise, its profit consists of sales revenue, surplus revenue, product cost, loss of stock loss and part of the supplier cost borne. The expected profit function of the core enterprise is:


$$\pi_{t}\left(q\right)=ps\left(q\right)+vI\left(q\right)-wQ-c_{u}L\left(q\right)-scQ=ps\left(q\right)+vQ-vs\left(q\right)-wQ-c_{u}\left(E\left(x\right)-s\left(q\right)\right)-scQ$$ (21)

$$\pi_{t}\left(q\right)=\left(p-v+c_{u}\right)s\left(q\right)+\left(v-w-sc\right)Q-c_{u}E\left(x\right)$$ (22)

The profit of the supplier is deter mined by the sales revenue and the remaining material cost excluding the core enterprise, so the expected profit function of the supplier is:


$$\pi_{s}\left(q\right)=wQ-\left(1-s\right)cQ$$ (23)

The optimal order quantity is still determined by the core enterprise, and the first order derivative of the expected profit of the core enterprise function can be obtained:


$$\frac{d\pi_{t}\left(q\right)}{dQ}=\left(p-v+c_{u}\right)*\left(1-F\left(q\right)\right)+v-w-sc$$ (24)

$$\frac{d\pi_{t}\left(q\right)}{dQ}=p+c_{u}-w-sc-\left(p-v+c_{u}\right)F\left(q\right)$$ (25)

Continue to find the second-order derivative of the function:


$$\frac{d^{2}\pi_{t}\left(q\right)}{dQ^{2}}=-\left(p-v+c_{u}\right)f\left(q\right)<0$$ (26)

Therefore, the second partial derivative of the expected profit function of the core enterprise is still less than 0, and the function is a convex function about q, so there is also a unique optimal order quantity that maximizes the expected profit.


$$\frac{d\pi_{t}\left(q\right)}{dQ}=p+c_{u}-w-sc-\left(p-v+c_{u}\right)F\left(q\right)=0$$ (27)

$${Q_{1}}^{*}=F^{-1}\left(\frac{p+c_{u}-w-sc}{p+c_{u}-v}\right)$$ (28)

For suppliers, his expected profit can be obtained according to the order volume of the core enterprise.

From the perspective of supply chain management, the part of the material cost only flows from the supplier to the core enterprise, and the overall profit of the supply chain will not increase or decrease. Therefore, the overall expected profit of the supply chain is still:


$$\pi \left(q\right)=\pi_{t}\left(q\right)+\pi_{s}\left(q\right)=\left(p-v+c_{u}\right)s\left(q\right)+\left(v-c\right)Q-c_{u}E\left(x\right)$$ (29)

As can be seen from the above, the corresponding order quantity of the supply chain is:


$${Q_{2}}^{*}=F^{-1}\left(\frac{p+c_{u}-c}{p-v+c_{u}}\right)$$ (30)

Obviously, therefore:


$$\frac{p+c_{u}-w-sc}{p+c_{u}-v}=\frac{p+c_{u}-c}{p-v+c_{u}}$$ (31)

w=(1−s)c (32)

Therefore, the overall coordination of the supply chain and the profit maximization can be realized by adjusting the cost distribution ratio and the material selling price of suppliers.

### 3.6. Research on supply chain financing decision-making coordination under revenue sharing contract

**Fig 4:**
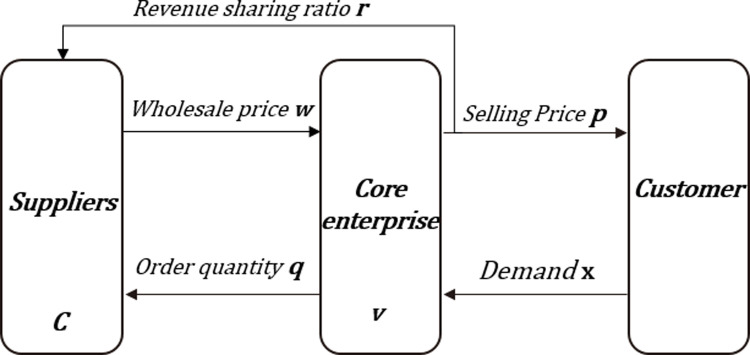
Revenue-sharing model of the supply chain.

As shown in Fig 4, according to the double marginal benefit in the supply chain, the benefit sharing contract model of the construction industry is constructed to coordinate the conflict of interest in the supply chain. The revenue-sharing contract requires the contractor to sacrifice part of the proceeds to the supplier, who will provide the contractor with a lower sale price to supplement the contractor’s losses. Core enterprises negotiate with suppliers on the income distribution ratio and reach an agreement. The retailer determines the optimal order quantity, and after the cycle ends, the retailer processes the remaining goods and transfers the r portion of its own sales revenue to the suppliers[10].

For the core enterprise, its profit consists of the rest of sales revenue, surplus income, product cost and loss of stock, so the expected profit function of the core enterprise is as follows:


$$\pi_{t}\left(q\right)=\left(1-r\right)ps\left(q\right)+vI\left(q\right)-wq-c_{u}L\left(q\right)=ps\left(q\right)+vq-vs\left(q\right)-wq-c_{u}\left(E\left(x\right)-s\left(q\right)\right)$$ (33)

$$\pi_{r}\left(q\right)=\left(1-r)ps\left(q\right)+(c_{u}-v\right)s\left(q\right)+\left(v-w\right)Q-c_{u}E\left(x\right)$$ (34)

For the supplier, its profit is composed of its own sales revenue, part of the contractor’s sales revenue and material cost, so the supplier’s expected profit function is:


$$\pi_{s}\left(q\right)=wQ+tps\left(q\right)-cQ$$ (35)

The optimal order quantity of the core enterprise is determined by the expected profit function, and the partial derivative of the core enterprise profit function can be derived according to the Leibzny formula:


$$\frac{d\pi_{t}\left(q\right)}{dQ}=\left(\left(1-t\right)p-v+c_{u}\right)*\left(1-F\left(q\right)\right)+v-w=\left(1-t\right)p+c_{u}-w-\left(\left(1-t\right)p-v+c_{u}\right)F\left(q\right)$$ (36)

Considering the actual situation, even if the sales price is divided, the price should still be greater than the residual value,


$$\frac{d^{2}\pi_{t}\left(q\right)}{dQ^{2}}=-\left(\left(1-t\right)p-v+c_{u}\right)f\left(q\right)<0$$ (37)

Therefore, the second partial derivative of the expected profit function of the core enterprise is less than 0, and the function is a convex function about q, so there is also a unique optimal order quantity that maximizes the expected profit.


$$\frac{d\pi_{t}\left(q\right)}{dQ}=\left(1-t\right)p+c_{u}-w-\left(\left(1-t\right)p-v+c_{u}\right)F\left(q\right)=0$$ (38)

$${Q_{1}}^{*}=F^{-1}\left(\frac{\left(1-t\right)p+c_{u}-w}{\left(1-t\right)p-v+c_{u}}\right)$$ (39)

Suppliers also determine their own profits according to the optimal order quantity, and can substitute the optimal order quantity into the expected profit function of the core enterprises and suppliers, and the overall profit of the supply chain can be obtained.

From the perspective of supply chain management, the part of sales revenue only flows from the core enterprise to the suppliers, and the overall profit of the supply chain remains unchanged, so the overall expected profit of the supply chain is still:


$$\pi \left(q\right)=\pi_{t}\left(q\right)+\pi_{s}\left(q\right)=\left(p-v+c_{u}\right)s\left(q\right)+\left(v-c\right)Q-c_{u}E\left(x\right)$$ (40)

As can be seen from the above, the corresponding order quantity of the supply chain is:


$${Q_{2}}^{*}=F^{-1}\left(\frac{p+c_{u}-c}{p-v+c_{u}}\right)$$ (41)

In order to coordinate the overall revenue of the supply chain to reach the centralized profit level of the centralized supply chain, because the distribution function is a monotonous function, so, namely: ${{\text{Q}}_{1}}^{\text{*}}={{\text{Q}}_{2}}^{\text{*}}$


$$\frac{\left(1-t\right)p+c_{u}-w}{\left(1-t\right)p-v+c_{u}}=\frac{p+c_{u}-c}{p-v+c_{u}}$$ (42)

$$w=c-\frac{tp\left(c-v\right)}{p+c_{u}-v}$$ (43)

Therefore, the overall coordination of the supply chain and profit maximization can be realized by adjusting the ratio of revenue and the material price of suppliers.

### 3.7. Study on supply chain financing decision coordination under cost sharing-benefit sharing contract

**Fig 5:**
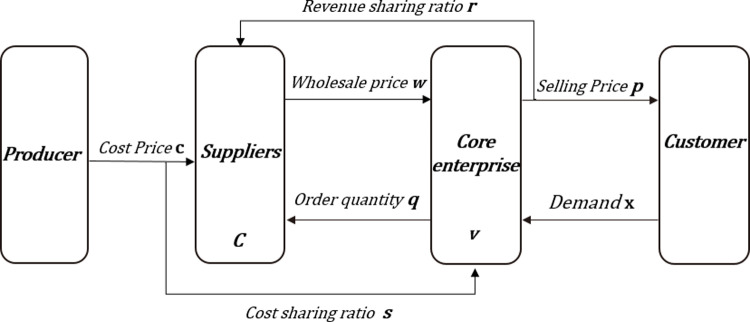
Combined contract model of the supply chain.

Based on this, this section discusses the combined contract model of cost sharing-benefit sharing. Fig 5 represents the supply chain model with contracts. The combined model of cost sharing and revenue sharing combines the characteristics of two cooperation modes of cost sharing and revenue sharing [11]. First, it not only encourages suppliers to reduce project costs by reducing costs and improving efficiency, but also encourages core enterprises to achieve revenue growth by maximizing project profits. Both sides have common incentives to achieve their common goals, which helps to improve the efficiency and results of cooperation. Second, the cost sharing part ensures that the risks are shared to some extent, while the revenue sharing part ensures that both parties share the benefits of the success of the project. This balance of risk and return helps build a solid partnership while reducing the pressure to take risk unilaterally. Third, such a model can be adjusted and adapted according to the characteristics of different projects and the actual situation of both partners. The flexible contract structure enables both parties to better respond to market changes and project needs, and improve the competitiveness and profit level of both parties [12].

The expected profits of the core enterprises similar to the previous two sections are:


$$\pi_{t}\left(q\right)=\left(1-r)ps\left(q\right)+(c_{u}-v\right)s\left(q\right)+\left(v-w-sc\right)Q-c_{u}E\left(x\right)$$ (44)

The expected profit of the supplier is:


$$\pi_{s}\left(q\right)=wQ+tps\left(q\right)-\left(1-s\right)cQ$$ (45)

The first-order derivative of the core enterprise profit function is:


$$\frac{d\pi_{t}\left(q\right)}{dQ}=\left(1-t\right)p+c_{u}-w-sc-\left(\left(1-t\right)p-v+c_{u}\right)F\left(q\right)=0$$ (46)

$${Q_{1}}^{*}=F^{-1}\left(\frac{\left(1-t\right)p+c_{u}-w-sc}{\left(1-t\right)p-v+c_{u}}\right)$$ (47)

The corresponding order quantity of the supply chain is:


$${Q_{2}}^{*}=F^{-1}\left(\frac{p+c_{u}-c}{p-v+c_{u}}\right)$$ (48)

In order to coordinate the overall revenue of the supply chain to reach the centralized profit level of the centralized supply chain, because the distribution function is a monotonous function, so, namely: ${{\text{Q}}_{1}}^{\text{*}}={{\text{Q}}_{2}}^{\text{*}}$


$$\frac{\left(1-t\right)p+c_{u}-w-sc}{\left(1-t\right)p-v+c_{u}}=\frac{p+c_{u}-c}{p-v+c_{u}}$$ (49)

$$w=\left(1-s\right)c-\frac{tp\left(c-v\right)}{p+c_{u}-v}$$ (50)

In this case, the overall coordination of the supply chain and profit maximization are realized by adjusting the distribution ratio of costs and benefits and the material price of suppliers.

## 4. Numerical analysis

### 4.1. Parameter settings

To facilitate analysis, this paper excludes the impact of external market uncertainties on the model.For convenient analysis, building materials random market demand obey the average of 500, standard deviation of 100 normal distribution, suppliers of unit building materials cost is 50 yuan, suppliers of building materials sales price is 70 yuan, the core enterprise unit building materials sales price is 100 yuan, the core unit of building materials loss of 30 yuan, the residual value of residual material is 20 yuan. The input parameters of the model are: $\text{x}\sim \text{N}\left(500,100\right),\text{c}=50,\text{w}=70,\text{p}=100,{\text{c}}_{\text{u}}=30,\text{v}=20$.

### 4.2. No contract situation

When the core enterprises and suppliers do not reach contractual cooperation, the overall expected profit of the supply chain is low, indicating that the overall optimization of the supply chain cannot be achieved only by the optimization of the core enterprises and suppliers respectively. Table 2 is an analysis of earnings without contract.

**Table 2:** Analysis table of earnings without contract.

	$\begin{array}{c} Q^{*} \end{array}$	$\begin{array}{c} \pi_{t}(q) \end{array}$	$\begin{array}{c} \pi_{s}\left(q\right) \end{array}$	$\begin{array}{c} \pi \left(q\right) \end{array}$
No contract circumstances	511.4	10665.19	10228.37	20893.56

### 4.3. Overall optimum situation of the supply chain

When the supply chain is optimal as a whole, the expected profit of the core enterprises and the expected profit of the suppliers are increased, which shows that the overall efficiency can be improved by coordinating all links of the supply chain. Table 3 shows the benefit analysis of the overall optimization of the supply chain.

**Table 3:** Benefit analysis table for the overall optimum situation of the supply chain.

	$\begin{array}{c} Q^{*} \end{array}$	$\begin{array}{c} \pi_{t}\left(q\right) \end{array}$	$\begin{array}{c} \pi_{s}\left(q\right) \end{array}$	$\begin{array}{c} \pi \left(q\right) \end{array}$
Supply chain optima	560.5	10172.08	11209.17	21381.25

### 4.4. Cost-sharing situation

Considering different cost-sharing ratio s   , suppliers will sell their building materials to core companies at a lower price. However, regardless of how the cost-sharing ratio is adjusted, the expected profit of the supply chain remains unchanged, and the expected profit of the supplier is 0. As shown in Table 4, this indicates that the cost-sharing strategy does not motivate the supplier under the current parameter setting, and other strategies are needed to improve the profit of the supplier.

**Table 4:** Benefit analysis table for the overall optimum situation of the supply chain.

$\begin{array}{c} s \end{array}$	$\begin{array}{c} w \end{array}$	$\begin{array}{c} Q^{*} \end{array}$	$\begin{array}{c} \pi_{t}\left(q\right) \end{array}$	$\begin{array}{c} \pi_{s}\left(q\right) \end{array}$	$\begin{array}{c} \pi \left(q\right) \end{array}$
0.1	45	560.5	21381.25	0	21381.25
0.2	40	560.5	21381.25	0	21381.25
0.3	35	560.5	21381.25	0	21381.25

### 4.5. Revenue-sharing situation

Considering different revenue sharing ratio r, with the increase of the revenue sharing ratio, the expected profit of the suppliers gradually increases, and the expected profit of the core enterprises gradually decreases, but the overall expected profit of the supply chain remains unchanged[13]. This shows that the revenue sharing strategy can realize the balance of suppliers and core enterprises by adjusting the sharing ratio, thus promoting cooperation.

According to the analysis of Table 5 and Table 6, when the revenue sharing ratio is between 0.2 and 0.4, the expected profit of both the core enterprise and the supplier is close to higher than that of the non-contract model, and the expected profit of the whole supply chain is also higher than that of the non-contract model. When the revenue sharing ratio is 0.7 or above, the core enterprise profit begins to be negative, which is impossible and therefore not considered. When the revenue sharing ratio is between 0.25 and 0.35, we find that the expected profit of both the core enterprise and the supplier is higher than that of the non-contract model when the revenue sharing ratio is between 0.31 and 0.324, and the expected profit of the supply chain is higher than that of the non-contract model. Therefore, by adopting the revenue sharing strategy and adjusting the revenue sharing ratio within this range, supply chain coordination can be realized and the expected profits of core enterprises and suppliers can be improved.

**Table 5:** Income analysis table under revenue sharing circumstances.

$\begin{array}{c} r \end{array}$	$\begin{array}{c} Q^{*} \end{array}$	$\begin{array}{c} \pi_{t}\left(q\right) \end{array}$	$\begin{array}{c} \pi_{s}\left(q\right) \end{array}$	$\begin{array}{c} \pi \left(q\right) \end{array}$
0.1	560.5	18073.86	3307.38	21381.25
0.2	560.5	14766.47	6614.77	21381.25
0.3	560.5	11459.09	9922.16	21381.25
0.4	560.5	8151.70	13229.54	21381.25
0.5	560.5	4844.319	16536.93	21381.25
0.6	560.5	1536.93	19844.31	21381.25
0.7	560.5	-1770.45	23151.70	21381.25
0.8	560.5	-5077.84	26459.09	21381.25

**Table 6:** Income analysis table under revenue sharing circumstances.

$\begin{array}{c} r \end{array}$	$\begin{array}{c} w \end{array}$	$\begin{array}{c} Q^{*} \end{array}$	$\begin{array}{c} \pi_{t}\left(q\right) \end{array}$	$\begin{array}{c} \pi_{s}\left(q\right) \end{array}$	$\begin{array}{c} \pi \left(q\right) \end{array}$
0.25	43.2	560.5	13112.79	8268.46	21381.25
0.3	41.8	560.5	11459.09	9922.16	21381.25
0.31	41.5	560.5	11128.35	10252.89	21381.25
0.316	41.4	560.5	10929.91	10451.34	21381.25
0.318	41.3	560.5	10863.76	10517.49	21381.25
0.32	41.2	560.5	10797.62	10583.63	21381.25
0.322	41.2	560.5	10731.47	10649.78	21381.25
0.324	41.2	560.5	10665.32	10715.93	21381.25
0.326	41.1	560.5	10599.17	10782.08	21381.25
0.328	41.1	560.5	10533.02	10848.23	21381.25
0.33	41.0	560.5	10466.88	10914.38	21381.25
0.34	40.7	560.5	10136.14	11245.11	21381.25
0.35	40.5	560.5	9805.40	11575.85	21381.25

**Fig 6:**
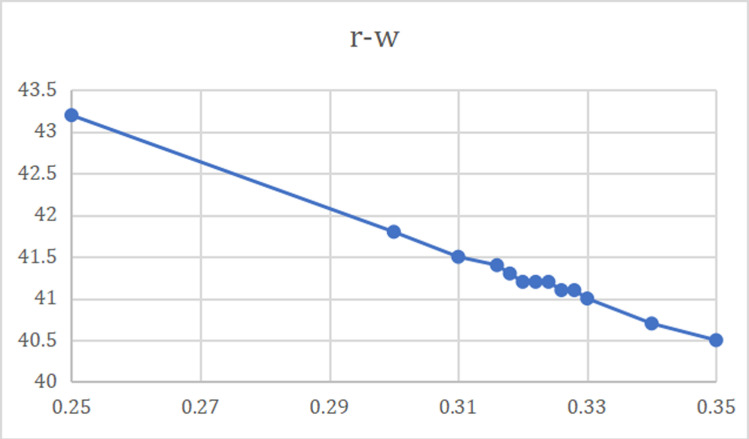
Relationship between revenue-sharing ratio and supply price.

**Fig 7:**
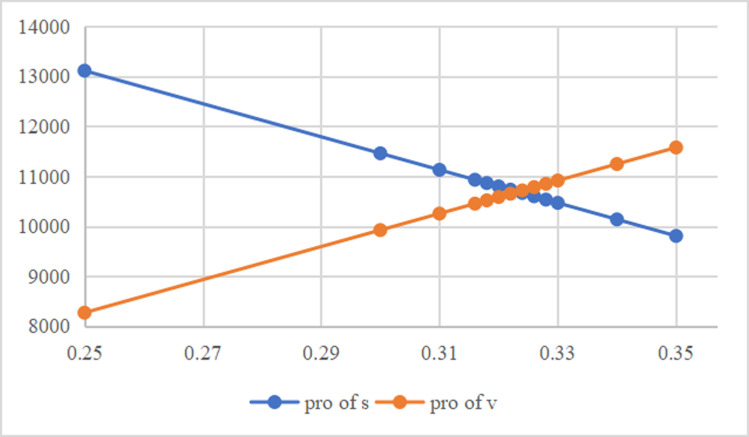
Relationship between revenue sharing ratio and Supplier profit and Core enterprise profit.

As can be seen from the Fig 6 and Fig 7, with the increase of the revenue sharing ratio, the supply price shows a linear downward trend. This means that core companies are willing to pay lower supply prices when revenue sharing is high. Suppliers accept lower supply prices by receiving more revenue share. In the range of revenue ratio of 0.31 to 0.35, although the supply price is declining, suppliers can obtain higher profits through sharing due to the increased revenue sharing ratio.

## 5. Conclusion

The financing coordination of construction supply chain is a complex and key issue. By discussing the financing coordination model of construction supply chain without contract and contract environment, this paper studies the interest game and cooperation mechanism between core enterprises, suppliers and banks[14].

In the non-contract environment, through the Stackelberg game model, the core enterprises and suppliers pursue the maximization of their own interests, this paper shows the cooperation problem between the core enterprises and the suppliers, as well as the overall profit optimization problem of the supply chain. Under the contract of cost sharing, both parties reduce the cost of the whole supply chain by jointly bearing the cost and maximizing the profit of the supply chain. Under the profit sharing contract, the core enterprises and suppliers encourage the cooperation by sharing the profits to maximize the overall profit of the supply chain. At the same time, the combined contract model of cost sharing and benefit sharing promotes the stable development of the cooperative relationship in the balance of risk and return, and realizes the maximization of the coordination and benefit of the supply chain.

In conclusion, the research in this paper not only provides a theoretical basis for the coordination of supply chain financing in the construction industry, but also provides useful insights for corporate decision-making in practice. Future research can further explore the implementation effects under different types of contractual models, combined with practical case studies, in order to better guide the financing decision-making and coordination management of the supply chain in the construction industry[15]. It is suggested that enterprises should pay more attention to supply chain cooperation and coordination in practice, establish long-term and stable cooperative relationships, and jointly cope with market challenges to achieve win-win development. May the research on supply chain financing coordination in the construction industry inject new impetus into the development of the industry, promote the continuous improvement of supply chain management in the construction industry, and promote the sustainable development of the whole industry.

## Supporting information

S1 Data Source Data. (ZIP)
